# Prevalence, intensity of infection and associated risk factors of soil-transmitted helminth infections among school children at Tachgayint woreda, Northcentral Ethiopia

**DOI:** 10.1371/journal.pone.0266333

**Published:** 2022-04-08

**Authors:** Tahir Eyayu, Gashaw Yimer, Lemma Workineh, Tegenaw Tiruneh, Meslo Sema, Biruk Legese, Andargachew Almaw, Yenealem Solomon, Birhanemaskal Malkamu, Ermias Sisay Chanie, Dejen Getaneh Feleke, Melkamu Senbeta Jimma, Seada Hassen, Aragaw Tesfaw

**Affiliations:** 1 Department of Medical Laboratory Sciences, College of Health Sciences, Debre Tabor University, Debre Tabor, Ethiopia; 2 Department of Pediatrics and Child Health Nursing, College of Health Sciences, Debre Tabor University, Debre Tabor, Ethiopia; 3 Department of Nursing, Faculty of Health Science, Assosa University, Assosa, Ethiopia; 4 Department of Environmental Health, College of Medicine and Health Science, Wollo University, Dessie, Ethiopia; 5 Department of Public Health, College of Health Sciences, Debre Tabor University, Debre Tabor, Ethiopia; UCSI University, MALAYSIA

## Abstract

**Background:**

Soil-transmitted helminths (STH) are one of the most common infections affecting underprivileged populations in low- and middle-income countries. *Ascaris lumbricoides*, *Trichuris trichiura*, and hookworm are the three main species that infect people. School children are the most vulnerable groups for STH infections due to their practice of walking and playing barefoot, poor personal hygiene, and environmental sanitation. However, evidence is limited in the study area. So, this study aimed to assess the current prevalence, infection intensity, and associated risk factors of STHs among school children in Tachgayint woreda, Northcentral Ethiopia.

**Methods:**

A cross-sectional study was conducted among school children of Tachgayint woreda from February to May 2021. The study participants were chosen via systematic random sampling. Stool samples were collected from 325 children and examined using the Kato-Katz technique. The data was analyzed using SPSS version 23. Binary and multivariable logistic regression analyses were used to identify the potential associated factors for STHs. An adjusted odds ratio (AOR) with a 95% confidence interval (CI) was used to measure the magnitude of the association. A P-value <0.05 was considered statistically significant.

**Results:**

The overall prevalence of STHs in this study was 36.0% (95% CI: 30.5–41.2%). *Ascaris lumbricoides* are the most prevalent species 89 (27.4%) followed by hookworm 14 (4.3%) and *Trichuris trichiura* 10 (3.1%). All of the infected school children had light-intensity of infections with the mean of eggs per gram (EPG) being 464.53. Lack of shoe wearing habit (AOR = 4.08, 95% CI: 1.29–12.88) and having untrimmed fingernail (AOR = 1.85, 95% CI: 1.06–3.22) were identified as risk factors for STH infections.

**Conclusions:**

More than one-third of the school children were infected with at least one STH species and this indicates that STHs are still a health problem among school children in the study area. Therefore, periodic deworming, implementation of different prevention strategies, and health education programs should be regularly applied in the area.

## Background

Soil-transmitted helminths (STHs) are a group of neglected tropical diseases that causes the most common infections worldwide and primarily affect marginalized populations in low- and middle-income countries. The three main species that infect people are *Ascaris lumbricoides (A*. *lumbricoides)*, *Trichuris trichiura (T*. *trichiura)*, and hookworms [1, 2]. More than 24% of the world’s population are infected with STHs infections with the greatest numbers of cases occurring in tropical and subtropical regions of Africa, Latin Americas, and East Asia [1, 3]. The high prevalence of STHs in these regions is related to low socioeconomic status and favorable environmental conditions that facilitate the transmission of STHs [4].

According to a 2010 survey, the global prevalence of *A*. *lumbricoide* was the most prevalent STHs infecting more than 819 million people followed by *T*. *trichiura* infecting 464 million people and hookworm which infects nearly 439 million people throughout the world. Furthermore, STHs are expected to cause 5.18 million disabled-adjusted life-years worldwide [5].

Based on a World Health Organization (WHO) fact sheet, 568 million School-Age Children (SAC) live in STHs transmitted areas, which shows that SAC are at higher risk of being infected with STHs [1]. This may be due to high exposure of children to contaminated soil when they play, walk barefoot, eat soil, poor personal hygiene, and environmental sanitation [6]. STHs are responsible for chronic infections, which may lead to school absenteeism, delayed physical growth, impaired cognitive development, and psychometrically performance of SAC [7].

Sub-Saharan African (SSA) countries have the greatest concentration of poverty in the world with a high prevalence of STHs infection. Between one-quarter and one-third of SSA’s population is affected by one or more STHs infection, especially school-aged children, disproportionately affected. Of the estimated 181 million school-aged children in SSA, almost one-half (89 million) are infected with hookworm, ascariasis, trichuriasis, or some combination of these STHs infections [8]. Ethiopia is one of the SSA countries with the second-highest burden of *A*. *lumbricoides*, the third-highest burden of hookworm, and the fourth-highest burden of *T*. *trichiura* infections in SSA [9].

In Ethiopia, STHs are the main and major public health problem. The national neglected tropical diseases mapping estimated that 79 million people are living in STHs endemic areas, which comprises 25.3 million school-aged children [10]. A recent meta-analysis revealed that the pooled prevalence of STHs infection among SAC of Ethiopia was 33.4%. A high prevalence of STHs was observed in Oromia (42.5%), followed by Southern Nations, Nationalities, and Peoples’ Region (SNNPR) (38.3%) and Amhara (32.9%) regional states. Species-based prevalence showed that *A*. *lumbricoides* was the most dominant STHs followed by *T*. *trichiura*, and hookworm with 19.9%, 12.4%, and 7.9% of infection, respectively, in the country [6].

In the presence of many prevention and control strategies, STHs are a widespread health problem in developing countries [2]. Therefore, close follow-up and regular STHs infection surveys are very important to update and know the prevalence of infection and effectiveness of implemented control measures.

However, there is limited evidence on the prevalence and intensity of STHs among schoolchildren in Tachgayint woreda, South Gondar Zone. Furthermore, assessing the current prevalence, infection intensity and identifying the associated risk factors of STHs infection is vital to guide implementers, public health planners, stakeholders, and policymakers to plan and design precise intervention strategies to eliminate STHs. Therefore, this study aimed to assess the current prevalence, infection intensity, and identify the associated risk factors for STHs among school children of Tachgayint woreda, South Gondar Zone, Northcentral Ethiopia.

## Methods and materials

### Study area

The study was conducted at Tateklesera elementary school. The school is located in Arebegbeya town, Tachgayint woreda, South Gondar Zone in Amhara National Regional State, Northcentral Ethiopia, which is located 776 km Northwest of Addis Ababa (capital city of Ethiopia) and 205 km from Bahir Dar (capital of Amhara region). The study area has altitudinal ranges from 750 to 2800 meters above sea level with 11° 45’ N and 38° 20’ E geographical coordinates. The climatic condition is “Woyna dega” with a mean annual minimum temperature of 17°C and a maximum of 25°C. According to the population projection of Ethiopia in 2017, an estimated total population of Tachgayint woreda was 116,876, of whom 58,273 were males and 58,603 were females [11].

### Study design, period, and population

A school-based cross-sectional study was conducted among school children of Tateklesera elementary school of Tachgayint woreda from February 1 to May 15, 2021. The school children who were willing to participate and provide sufficient stool samples were included, while the students who had taken anti-intestinal parasite drug/s within four weeks before data collection were excluded from the study.

### Sample size determination and sampling technique

The required sample size was calculated using a single population proportion formula;

$$n=\frac{{\left(Z_{\frac{\alpha }{2}}\right)}^{2}p\left(1-p\right)}{d^{2}}$$

where; n is the required sample size, Z*α/*_*2*_ *is the* level of confidence, p is the estimated prevalence of STH among school children, d is margin of error. The following assumptions were made to determine sample size; 95% level of confidence, 5% margin of error, 25.78% prevalence of STHs among school children in western Ethiopia [12] and with a 10% non-response rate. Hence, a total of 325 school children were included in this study.

In 2021, there were approximately 1390 students registered from grade 1 to 8 at Tateklesera elementary school. The students were stratified according to their educational levels (grade 1 to grade 8). There were 8 strata based on educational level. Then, the number of children sampled from each stratum was proportionally allocated by dividing the number of students in the stratum by the total number of students in the school, and finally, the proportion was multiplied by the total sample size (see Table 1). The K^th^ interval was determined by dividing the total number of students in the school by total sample size, and the K^th^ interval value is 4. Then, the first study participant student from each stratum was selected by using a systematic random sampling technique and the next study participant was selected every K^th^ interval by using a class roster.

**Table 1:** Total number of registered Tateklesera elementary school students in Northcentral Ethiopia by grade, and number of proportional sample size taken at each grade level for the study, February 1 to May 15, 2021.

Grade Level	Total students	Selected for the Study
1^st^	128	30
2^nd^	120	28
3^rd^	124	29
4^th^	137	32
5^th^	158	37
6^th^	163	38
7^th^	167	39
8^th^	393	92
**Total**	**1390**	**325**

### Data collection and processing

#### Questionnaire survey

The school children who participated in this study were informed about the purpose of the study. Face-to-face interviews with schoolchildren and their parents were conducted to collect sociodemographic information about study participants as well as associated factors for STHs. A pretested structured questionnaire which is written in the mother tongue (Amharic language) was used for data collection. During the interview, the fingernail status of the study participants was collected through direct observation.

#### Sample collection and laboratory analysis

A clean, dry, and leakproof container that was labeled with a unique identification number was given to collect the sample. About 5gm of stool specimen was collected from each selected study participant. The fresh stool samples were examined by direct wet mount microscopy and the Kato-Katz technique. Three Medical laboratory technologists perform the double Kato-Katz slides for the identification and count of STH eggs through a light microscope.

*Kato-Katz technique*. Used for qualitative and semi-quantitative diagnosis of intestinal helminthic infections. Feces were pressed through a mesh screen to remove large particles. A portion of the sieved sample is then transferred to the hole of a template holding 41.7 mg on a slide. After filling the hole, the template was carefully removed and the remaining sample was covered with a piece of cellophane soaked in glycerol. The glycerol clears the fecal material from around the eggs. Finally, the slides were examined under the microscope and the eggs were then counted for positive slides [13].

Kato-Katz slides were examined within one hour of its preparation for detection and counting of hookworm ova. Identification of other STHs also performed after one day. The total number of eggs were expressed as eggs per gram (EPG) of stool. EPG was calculated to classify the infection intensity as light, moderate, and heavy infection. The severity of STHs infection is defined as light, moderate, and heavy intensity of infections, respectively, as follows *A*. *lumbricoides*: 1 to 4999 EPG, 5000 to 49999 EPG, and ≥50000 EPG; hookworm: 1 to 1999 EPG, 2000 to 3999 EPG, and ≥4000 EPG; *T*. *trichiura*: 1 to 999 EPG, 1000 to 9999 EPG, and ≥10000 EPG [14].

### Data quality control

Firstly, the data collection tool (questionnaire) was prepared in the English language. Then, it was converted into the Amharic language. Lastly, it was retranslated back to English to retain its accuracy and consistency. Before data collection, training was given for data collectors and laboratory technologists. Two slides were prepared from each sample. The slides were examined by two different Medical Laboratory Technologists independently. The results of the microscopic examination were compared and discordant results were immediately fixed by cross-checking the slide by a senior medical parasitologist. And also, the mean of two slides egg count was used to classify infection intensity. Furthermore, all standard operating procedures were strictly followed during sample collection, processing, and examination to ensure the test result’s quality and reliability.

### Data management and analysis

Data were coded and entered into Epi Data Manager version 4.4.2.1 statistical software and then exported to SPSS version 23 for analysis. Descriptive statistics were done to summarize the sociodemographic characteristics of the study participants. The mean and standard deviation (SD) were calculated for parasite egg count. Binary logistic regression analyses were used to assess the association between the independent and dependent variables. Firstly, binary logistic regression analysis was done, and then to control the possible confounding, variables with a p-value < 0.2 were adjusted by using multivariable logistic regression analysis. The strength of the association between predictor and outcome variables were assessed by using the adjusted odds ratio (AOR) and 95% confidence interval (CI). In all cases, p-value < 0.05 was considered as a statistically significant association.

### Ethical consideration

The research was conducted after obtaining an ethical clearance letter from Debre Tabor University, College of Health Sciences Research and Ethical Review Committee (reference no. CHS/226/12 in Ethiopian calendar). A permission letter was obtained from the woreda health office and the director of the school. Written informed consent was obtained from each parent/legal guardian of the children. Information collected from the study participants was kept confidential. Any individuals who were positive for different STHs were linked to the responsible body for treatment. This study was conducted in accordance with the declaration of Helsinki.

## Result

### Socio-Demographic characteristics of study participants

A total of 325 school children participated in this study, with a response rate of 100%. Of these study subjects, 179 (55.1%) were females and 145 (44.9%) were males. The mean age of the study subjects was 12.03±2.4 years and the majority (53.5%) were in the age group of 11–14 years. A majority of the children were urban dwellers, 69.8%, and orthodox in religion (90.5%) (Table 2).

**Table 2:** Socio-demographic distribution of STH infections among school children of Tachgayint woreda, Northcentral Ethiopia, 2021.

Characteristics (n = 325)	Categories	STH infection		Total n (%)
		Positive, n (%)	Negative, n (%)	
**Sex**	Male	53 (16.3)	93 (28.6)	146 (44.9)
	Female	64 (19.7)	115 (35.4)	179 (55.1)
Age in years	≤ 10	36 (11.1)	53 (16.3)	89 (27.4)
	11–14	65 (20.0)	109 (33.5)	174 (53.5)
	≥15	16 ((4.9)	46 (14.2)	62 (19.1)
**Residence**	Urban	75(23.1)	152 (46.8)	227 (69.8)
	Rural	42 (12.9)	56 (17.2)	98 (30.2)
**Religion**	Orthodox	107 (32.9)	187 (57.5)	294 (90.5)
	Muslim	8 (2.5))	16 (4.9)	24 (7.4)
	Protestant	2 (0.6)	5 (1.5)	7 (2.2)
Grade	1–4	49 (15.1)	70 (21.5)	119 (36.6)
	5–8	68 (20.9)	138 (42.5)	206 (63.4)

### Prevalence and intensity of STH infections

**Fig 1:**
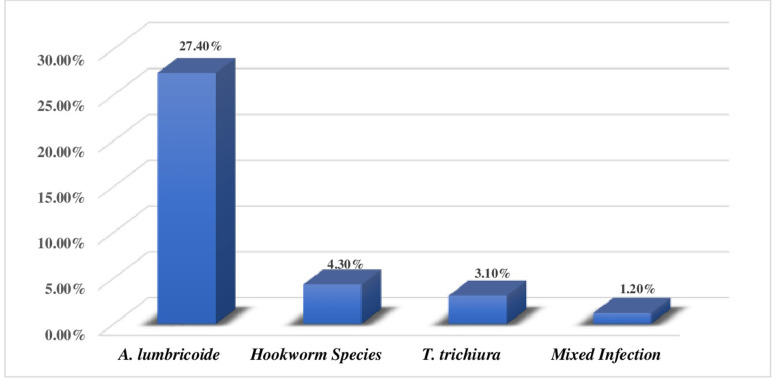
Prevalence of STH Species among school children of Tachgayint woreda, Northcentral Ethiopia, 2021.

The overall prevalence of STHs among school children was 36.0% (117/325) with a 95% CI of 30.5% - 41.2%. *Ascaris lumbricoides* were the most prevalent 89 (27.4%), followed by hookworm 14 (4.3%) and *T*. *trichiura* 10 (3.1%). Mixed STH infections were found only in 4 (1.2%) school children (Fig 1).

The overall mean of EPG of stool among STH-infected study participants was found to be 464.53 EPG of stool. The arithmetic means of fecal egg count of *A*. *lumbricoides* among infected children was 512.52 EPG (range: 72–1,560). For hookworm, it was 355.5 EPG (range: 96–720) and for *T*. *trichiura* the count was 238.0 EPG (range: 48–504).

Out of 117 school children who had STH infections, the intensity of infection with *A*. *lumbricoides*, hookworm, and *T*. *trichiura* was light among all infected study participants. In this study, there was no moderate or heavy intensity of infections caused by different species of STH (Table 3).

**Table 3:** Intensity of STHs infection among school children of Tachgayint woreda, Northcentral Ethiopia, 2021.

Type of STH infection	Total STH infected, n (%)	Infection Intensity	
		Light, n (%)	Mean EPG of stool (95% CI)
***A*. *lumbricoide***	93 (76.9)	93 (100)	512.52 (451.44–573.6)
**Hookworm**	16 (13.2)	16 (100)	355.5 (254.89–456.11)
***T*. *trichiura***	12 (9.9)	12 (100)	238.0 (150.65–325.35)
**Overall**	121 (100)	121 (100)	464.53 (412.95–516.11)

### Factors associated with STH infections among school children

Bivariable and multivariable logistic regression were used to determine the associated factors for STH infections. In this study, several risk factors were considered that contribute to STH infections (see Table 4). The potential associated factors such as handwashing habits after defecation, shoe-wearing habits, latrine utilization, and source of drinking water were found to be statistically associated with STH prevalence (p-value *<*0.05).

**Table 4:** Bivariable and multivariable logistic regression analysis of associated factors of STH infections among school children of Tachgayint woreda, Northcentral Ethiopia, 2021.

Variables		STHs Infection Status		COR (95% CI)	P-value	AOR (95% CI)	P-value
		Positive, n (%)	Negative, n (%)				
**Sex**	Male	53 (36.3)	93 (63.7)	1.024 (0.65, 1.61)	0.919		
	Female	64 (35.8)	115 (64.2)	1.0			
**Age in years**	≤ 10	36 (40.4)	53 (59.6)	1.95 (0.96, 3.97)	0.064	2.08 (0.65, 6.67)	0.219
	11–14	65 (37.4)	109 (62.6)	1.71 (0.90, 3.27)	0.102	2.06 (0.99, 4.30)	0.053
	≥15	16 (25.8)	46 (74.2)	1.0		1.0	
**Residence**	Urban	75(33.0)	152 (67.0)	1.0		1.0	
	Rural	42 (42.9)	56 (57.1)	1.52 (0.93, 2.47)	0.092	1.56 (0.92, 2.65)	0.099
**Grade**	1–4	49 (41.2)	70 (58.8)	1.42 (0.89, 2.26)	0.140	1.15 (0.50, 2.65)	0.744
	5–8	68 (33.0)	138 (67.0)	1.0		1.0	
**Contact with river**	Yes	65 (37.1)	110 (62.9)	1.11 (0.71, 1.75)	0.643		
	No	52 (34.7)	98 (65.3)	1.0			
**Wash cloth on river**	Yes	61 (37.9)	100 (62.1)	1.18 (0.75, 1.85)	0.482		
	No	56 (34.1)	108 (65.9)	1.0			
**Swimming on the river**	Yes	47 (36.7)	81 (63.3)	1.05 (0.66, 1.67)	0.828		
	No	70 (35.5)	127 (64.5)	1.0			
**Hand washing habit after defecation**	Always	63 (31.5)	137 (68.5)	1.0		1.0	
	Sometimes	41 (39.4)	63 (60.6)	1.41 (0.86, 2.32)	0.168	1.64 (0.93, 2.90)	0.087
	Not at all	13 (61.9)	8 (38.1)	3.53 (1.39, 8.95)	0.008	1.70 (0.58, 5.01)	0.336
**Hand washing habit before a meal**	Always	92 (36.4)	161 (63.6)	1.0			
	Sometimes	49 (65.3)	25 (34.7)	0.93 (0.54, 1.61)	0.798		
**Shoe wearing habit**	Always	87 (34.1)	168 (65.9)	1.0		1.0	
	Sometimes	12 (26.1)	34 (73.9)	0.68 (0.34, 1.38)	0.288	0.65 (0.28, 1.51)	0.314
	Not at all	18 (75.0)	6 (25.0)	5.79 (2.22, 15.12)	0.000	4.08 (1.29, 12.88)	0.016*
**Habit of latrine utilization**	Always	73 (38.6)	116 (61.4)	1.0		1.0	
	Sometimes	35 (28.9)	86 (71.1)	0.65 (0.40, 1.06)	0.081	0.65 (0.36, 1.17)	0.151
	Not at all	9 (60.0)	6 (40.0)	2.38 (0.81, 6.97)	0.113	1.15 (0.32, 4.18)	0.831
**Fingernail status**	T**r**immed	76 (31.1)	168 (68.9)	1.0		1.0	
	Not t**r**immed	41 (50.6)	40 (49.4)	2.27 (1.36, 3.78)	0.002	1.85 (1.06, 3.22)	0.031*
**Habit of eating raw vegetable**	Always	15 (44.1)	19 (55.9)	1.02 (0.49, 2.10)	0.962		
	Sometimes	52 (36.6)	90 (63.4)	0.81 (0.48, 1.36)	0.426		
	Not at all	23 (35.9)	41 (64.1)	1.0			
**Habit of eating raw meat**	Yes	29 (37.2)	49 (62.8)	1.09 (0.67–1.75)	0.732		
	No	61 (37.7)	101 (62.3)	1.0			
**Source of drinking water**	Pipe	90 (33.6)	178 (66.4)	1.0		1.0	
	Well	12 (41.4)	17 (58.6)	1.40 (0.64, 3.05)	0.403	1.67 (0.68, 4.08)	0.265
	Stream	15 (53.6)	13 (46.4)	2.28 (1.04, 5.00)	0.039	2.28 (0.98, 5.34)	0.057

Note: *p-value <0.05; AOR: Adjusted Odds Ratio; COR: Crude Odds Ratio; CI: Confidence Interval.

A multivariable logistic analysis was conducted after adjusting variables with a p-value *<*0.2 in the bivariable logistic analysis. The multivariable logistic regression model estimated that school children who had no shoe-wearing habits were 4 times (AOR = 4.08, 95% CI: 1.29–12.88; *P* = 0.016) more likely to be infected with STHs than school children who had the habit of shoe-wearing. The odds of STH infection were 1.8 times higher (AOR = 1.8, 95% CI: 1.06–3.22; *P* = 0.031) for school children who had not trimmed their fingernails as compared to those who had trimmed their fingernails (Table 4).

## Discussion

In this study, the overall prevalence of STH infections with at least one STH parasite was 36.0% (95% CI: 30.5% - 41.2%), which would be classified into the moderate risk areas category where prevalence is between 20% and 50%, and meet the requirements of STH mass drug administration once a year [2]. The overall prevalence of STH infections observed in this study is comparable with studies conducted in other parts of Ethiopia (Amhara region, Kerewo Town, West Shoa Zone, Northwest Ethiopia) [15–18], Tanzania [19], and Malaysia [20].

However, the STH prevalence in our study is slightly lower than the prevalence reported in Sigmo Primary School, South-Western Ethiopia (41.7%) [21] and Anambra State, Nigeria (42.2%) [22]. In addition, the prevalence in our study is significantly lower than the findings of other studies conducted in a different part of Ethiopia with the prevalence ranging from 52.0% - 67.9% [23–28] and western Kenya (44.05%) [29], Nigeria (44.2%) [30], Western Rwanda (77.7%) [31] and India (54.8%) [32]. On the other hand, the prevalence of STH infections in this study was higher than those of local studies conducted in Ethiopia, such as the prevalence of STH infections among school children of Southern Ethiopia (23.1%) [33], Sekela Primary School (25.78%) [12], Goro Primary School (15.8%) [34], Gurage zone (9.5%) [35], and Ambo town (12.6%) [36]. The differences in the prevalence of STH infections among different studies could be explained by variations in the study period, sample size, geographical location, urbanization, practices of personal hygiene, level of environmental sanitation, and source of drinking water. For instance, some of the above studies used different types of laboratory techniques like direct wet mount, formol-ether concentration technique, and McMaster diagnostic technique. Thus, dissimilarities in the diagnostic technique’s sensitivity might be the possible source of discrepancies in the prevalence of STH infections.

Furthermore, the current STH infections prevalence was higher than the studies conducted in different countries of the world, such as reports from African countries (Nigeria (17.1%) [37], Gabon (15.0%) [38], West Region of Cameroon (8.7%) [39], Togo (5.0%) [40]), Colombia (29.6%) [41], India (7.7% and 7.0%) [42, 43] and Thailand (3.13%) [44]. The possible reason for this inconsistency in the prevalence of STH infections might be due to the differences in socio-cultural determinants, behavioral characteristics, climatic conditions, implementation of prevention and control measures, and frequency and application of mass drug administration on intestinal parasites among different countries.

The most predominant STH infection identified in this study is *Ascaris lumbricoides* 27.4% with a 95% CI of (22.5% - 32.6%) followed by hookworm 4.3% with a 95% CI of (2.2% - 6.8%). This predominance was consistent with some studies conducted in our country [12, 18, 23, 36] and different parts of the world [22, 38] that indicated the dominant STH infections among schoolchildren were *A*. *lumbricoides* followed by hookworm. The reason behind the predominance of *A*. *lumbricoides* both in the current and previous studies could be related to the long life of the female worm and has a fecundity rate of about 134,000 to 360, 000 eggs per day for about 300 days. Consequently, vast numbers of eggs are discharged into the human environment daily. Moreover, the hard nature of the eggs of *A*. *lumbricoides* to resist adverse environmental conditions more than other STHs can contribute to sustaining the transmission cycle for a longer period [45].

The prevalence of *A*. *lumbricoides* infections observed in the present study was 27.4%. These were comparable with the studies conducted in Southwest and Northwest Ethiopia [18, 25]. But, the prevalence of *A*. *lumbricoides* was higher than the prevalence reported from the study conducted in Hawassa [33], Sekela [12], Ambo town [36], and Gabon (10.4%) [38]. On the contrary, it is lower than that reported from Southern Ethiopia [23, 24], Western Rwanda [31], and India [32]. The second most prevalent STH infection was hookworm (4.3%), which is similar to the report of South and Southcentral Ethiopia [27, 35]. This data is lower than the studies conducted in the Amhara region (20.6%) [15], West Shoa Zone (17.9%) [17], Northwest Ethiopia (8.9%) [18], Jimma town (11.6%) [25], Goro Primary School (10.07%) [34], and Nigeria (15.7%) [30]. However, it is higher than the previous reports from South-Western Ethiopia (1%) [21], western Kenya (0.27%) [29], and Thailand (3.13%) [44]. The prevalence of *T*. *trichiura* in the current study was 3.1% and similar results were reported from the Amhara region and Ambo town [15, 36]. However, it is lower than previous studies reported from Southwest Ethiopia [21, 25], Tanzania [19], and Gabon [38]. Level of poverty, environmental sanitary practices, implementation of different preventive and control measures, Climate variability, and geographical distribution of the parasites might have contributed to the infection rate differences across different regions. Moreover, the variation might be related to temperature differences and characteristics of the soil which helps for maturation of the non-infective stage of the parasite to infective one and easily transmission of the parasites [4].

Almost all positive cases with different species of STH infections were showing the light intensity of infection that is in line with the previous report in the Gurage zone, South Central Ethiopia [35]. But other previous studies showed the occurrences of the moderate and heavy intensity of infection [18, 24, 29, 33] other than light infection intensities; which could be the positive impact to validate for the elimination of STH infection in our country. This result is lower than the WHO elimination target of STHs, which is defined as a *<* 2% proportion of STH infections of moderate and heavy intensity due to *A*. *lumbricoides*, *T*. *trichuria*, and hookworm [46].

Moreover, the current study has identified potential risk factors for STHs infection among school children. Accordingly, shoe-wearing habits and fingernail status were found to be significant predictors of STH infection. Study participants, who lack the habit of shoe-wearing were 4 times more likely to be infected with STHs than school children who had regular shoe-wearing habits (AOR = 4.08, 95% CI: 1.29–12.88; P = 0.016). This was similar to other studies conducted in Sekela [12], Goro [34], and Kerewo Town [16] primary schools. This finding might be due to fact that hookworm infections were higher in children not wearing shoes. This indicates that wearing shoes has great importance in protecting against the transmission, contamination, and invasion of the infective stage of the parasite into the skin [34].

We also found that those students without the habit of nail trimming were more likely to be affected by STHs infection, which is in agreement with studies conducted in Ethiopia [17, 23, 36] and Thailand [44]. The possible reason might be due to the outdoor playing habits of school children on poor sanitation areas, which results in contamination of their hands. Dirt under fingernails may harbor different stages of parasites, which can be ingested during food eating and nail-biting or thumb sucking [23, 44].

## Limitations of the study

Due to budget constraints, this study did not focus on molecular assays and other techniques that are best to estimate the prevalence of STHs and differentiate different species of hookworm. Also, Harada-Mori technique and culture on agar plates were not used to detect *Strongyloid*es species, and it might have caused missing this species.

## Conclusions

More than one-third of the school children were infected with at least one STH species and this indicates that STHs are still a health problem among school children of Tachgayint woreda, Northcentral Ethiopia. Based on the WHO guideline, the study area would be classified into the moderate risk areas category, which needs STH mass drug administration once a year. The present study has also revealed that *A*. *lumbricoides* is the most prevalent species that cause infection in school children. Soil-transmitted helminth infections were strongly associated with shoes wearing habits and fingernail status of school children. Therefore, periodic deworming of school children, design and implementation of prevention and control strategies, and health education in study areas are very essential to decrease the burden of the disease.

## Supporting information

S1 Table Prevalence of STH species among school children of Tachgayint woreda, Northcentral Ethiopia. (DOCX)

## References

[1] Organization WH. Soil-transmitted helminth infections; 2021. Available from: https://wwwwhoint/news-room/fact-sheets/detail/soil-transmitted-helminth-infections. Accessed on December 2, 2021().

[2] Organization WH. Soil-transmitted helminthiases: eliminating as public health problem soil-transmitted helminthiases in children: progress report 2001–2010 and strategic plan 2011–2020. 2012.

[3] Montresor A, Mupfasoni D, Mikhailov A, Mwinzi P, Lucianez A, Jamsheed M, et al. The global progress of soil-transmitted helminthiases control in 2020 and World Health Organization targets for 2030. 2020;14(8):e0008505. 10.1371/journal.pntd.0008505 PMC7446869 32776942

[4] Brooker S, Clements AC, Bundy DA. Global epidemiology, ecology and control of soil-transmitted helminth infections. Advances in parasitology. 2006;62:221–61. doi: 10.1016/S0065-308X(05)62007-6 16647972 PMC1976253

[5] Pullan RL, Smith JL, Jasrasaria R, Brooker SJ. Global numbers of infection and disease burden of soil transmitted helminth infections in 2010. Parasites & vectors. 2014;7(1):37. doi: 10.1186/1756-3305-7-37 24447578 PMC3905661

[6] Hailegebriel T, Nibret E, Munshea AJIDR, Treatment. Prevalence of Soil-Transmitted Helminth Infection Among School-Aged Children of Ethiopia: A Systematic Review and Meta-Analysis. 2020;13:1178633720962812. 10.1177/1178633720962812 PMC7543112 33088182

[7] Pabalan N, Singian E, Tabangay L, Jarjanazi H, Boivin MJ, Ezeamama AEJPntd. Soil-transmitted helminth infection, loss of education and cognitive impairment in school-aged children: A systematic review and meta-analysis. 2018;12(1):e0005523. 10.1371/journal.pntd.0005523 PMC5766095 29329288

[8] Hotez PJ, Kamath A. Neglected tropical diseases in sub-Saharan Africa: review of their prevalence, distribution, and disease burden. PLoS Negl Trop Dis. 2009;3(8):e412. doi: 10.1371/journal.pntd.0000412 19707588 PMC2727001

[9] Deribe K, Meribo K, Gebre T, Hailu A, Ali A, Aseffa A, et al. The burden of neglected tropical diseases in Ethiopia, and opportunities for integrated control and elimination. Parasites & vectors. 2012;5(1):240. 23095679 10.1186/1756-3305-5-240 PMC3551690

[10] Negussu N, Mengistu B, Kebede B, Deribe K, Ejigu E, Tadesse G, et al. Ethiopia schistosomiasis and soil-transmitted helminthes control programme: progress and prospects. Ethiopian medical journal. 2017;55(Suppl 1):75. 28878432 PMC5582635

[11] Ababa A. Federal Democratic Republic of Ethiopia Central Statistical Agency Population Projection of Ethiopia for All Regions At Wereda Level from 2014–2017. Addis Ababa: Central Statistical Agency. 2014.

[12] Tolera A, Dufera MJJoPR. The prevalence of soil-transmitted helminths and associated risk factors among school children at Sekela Primary School, Western Ethiopia. 2020;2020. 10.1155/2020/8885734 PMC7648701 33194226

[13] Bosch F, Palmeirim MS, Ali SM, Ame SM, Hattendorf J, Keiser JJPntd. Diagnosis of soil-transmitted helminths using the Kato-Katz technique: What is the influence of stirring, storage time and storage temperature on stool sample egg counts? 2021;15(1):e0009032. 10.1371/journal.pntd.0009032 PMC7857572 33481808

[14] Organization WH. Prevention and control of schistosomiasis and soil-transmitted helminthiasis: report of a WHO expert committee. WHO technical report series 912.: WHO; 2002. Geneva, Switzerland. p. 12592987

[15] Nute AW, Endeshaw T, Stewart AE, Sata E, Bayissasse B, Zerihun M, et al. Prevalence of soil-transmitted helminths and Schistosoma mansoni among a population-based sample of school-age children in Amhara region, Ethiopia. 2018;11(1):1–9. 10.1186/s13071-018-3008-0 PMC6056938 30041691

[16] Yarinbab T, Darcha AJJIDE. Prevalence and determinants of soil transmitted helminthes infections among primary school children in Kerewo Town, Gena Bossa Woreda, Ethiopia: cross sectional study. 2019;5:90. doi: 10.1002/ijgo.12977 31541585 PMC7646192

[17] Ibrahim T, Zemene E, Asres Y, Seyoum D, Tiruneh A, Gedefaw L, et al. Epidemiology of soil-transmitted helminths and Schistosoma mansoni: a base-line survey among school children, Ejaji, Ethiopia. 2018;12(12):1134–41. 10.3855/jidc.9665 32027616

[18] Zeleke AJ, Derso A, Bayih AG, Gilleard JS, Eshetu TJR, Medicine RiT. Prevalence, Infection Intensity and Associated Factors of Soil-Transmitted Helminthiasis Among School-Aged Children from Selected Districts in Northwest Ethiopia. 2021;12:15. 10.2147/RRTM.S289895 PMC7894853 33623469

[19] Eltantawy M, Orsel K, Schroeder A, Morona D, Mazigo HD, Kutz S, et al. Soil transmitted helminth infection in primary school children varies with ecozone in the Ngorongoro Conservation Area, Tanzania. 2021;49(1):1–12. 10.1186/s41182-021-00310-6 PMC7945338 33691800

[20] Alaribi FI, Unyah NZ, Misni N, Masri SN, Osman MJMJoM, Sciences H. The Prevalence of Soil-Transmitted Helminths Infection and Its Association with Anaemia Among Refugee School Children in The Klang Valley, Malaysia. 2020;16(4).

[21] Emana D, Jemal K, Bajiro M, Mekonnen ZJCMR. Prevalence and intensity of soil-transmitted helminths among school-aged children in Sigmo Primary School, Jimma Zone, South-Western Ethiopia. 2015;4(4):98–103.

[22] Nwankwo A, Onyebueke AC, Irikannu KC, Nzeukwu CI, Onwuzulike IV, Okafor NM. Soil-Transmitted Helminths Infection and Associated Risk Factors among Primary School Pupils in Omogho and Awa Communities, Anambra State, Nigeria.

[23] Eyamo T, Girma M, Alemayehu T, Bedewi ZJR, medicine rit. Soil-transmitted helminths and other intestinal parasites among schoolchildren in Southern Ethiopia. 2019;10:137. doi: 10.2147/RRTM.S210200 31695554 PMC6817342

[24] Gebreyesus TD, Tadele T, Mekete K, Barry A, Gashaw H, Degefe W, et al. Prevalence, intensity, and correlates of schistosomiasis and soil-transmitted helminth infections after five rounds of preventive chemotherapy among school children in Southern Ethiopia. 2020;9(11):920. 10.3390/pathogens9110920 PMC7694749 33172114

[25] Mekonnen Z, Hassen D, Debalke S, Tiruneh A, Asres Y, Chelkeba L, et al. Soil-transmitted helminth infections and nutritional status of school children in government elementary schools in Jimma Town, Southwestern Ethiopia. 2020;8:2050312120954696. 10.1177/2050312120954696 PMC7475784 32953118

[26] Alelign T, Degarege A, Erko BJJopr. Soil-transmitted helminth infections and associated risk factors among schoolchildren in Durbete Town, Northwestern Ethiopia. 2015;2015. 10.1155/2015/641602 PMC4487929 26161265

[27] Hailu Amare H, Lindtjørn BJPntd. Helminth infections among rural schoolchildren in Southern Ethiopia: a cross-sectional multilevel and zero-inflated regression model. 2020;14(12):e0008002. doi: 10.1371/journal.pntd.0008002 33351816 PMC7755205

[28] Tadege B, Shimelis TJPo. Infections with Schistosoma mansoni and geohelminths among school children dwelling along the shore of the Lake Hawassa, southern Ethiopia. 2017;12(7):e0181547. 10.1371/journal.pone.0181547 PMC5515461 28719642

[29] Ngonjo T, Okoyo C, Andove J, Simiyu E, Lelo AE, Kabiru E, et al. Current status of soil-transmitted helminths among school children in Kakamega County, Western Kenya. 2016;2016. doi: 10.1155/2016/7680124 27525108 PMC4971323

[30] Omotola O, Ofoezie IJJBP. Prevalence and intensity of soil transmitted helminths among school children in Ifetedo, Osun State, Nigeria. 2019;10(352):2. doi: 10.1152/ajpendo.00365.2019 31689145 PMC6957379

[31] Kabatende J, Mugisha M, Ntirenganya L, Barry A, Ruberanziza E, Mbonigaba JB, et al. Prevalence, intensity, and correlates of soil-transmitted helminth infections among school children after a decade of preventive chemotherapy in Western Rwanda. 2020;9(12):1076. 10.3390/pathogens9121076 PMC7767502 33371488

[32] Gupta A, Acharya AS, Rasania SK, Ray TK, Jain SKJIJoPH. Prevalence and risk factors of soil-transmitted helminth infections in school age children (6–14 years)–A cross-sectional study in an urban resettlement colony of Delhi. 2020;64(4):333. 10.4103/ijph.IJPH_120_20 33318381

[33] Gitore WA, Ali MM, Yoseph A, Mangesha AE, Debiso ATJPo. Prevalence of soil-transmitted helminthes and its association with water, sanitation, hygiene among schoolchildren and barriers for schools level prevention in technology villages of Hawassa University: Mixed design. 2020;15(9):e0239557. doi: 10.1371/journal.pone.0239557 32970747 PMC7514018

[34] Tiruneh T, Geshere G, Ketema TJIJoP. Prevalence and determinants of soil-transmitted helminthic infections among school children at goro primary school, South West Shewa, Ethiopia. 2020;2020. 10.1155/2020/8612054 PMC7482002 32952576

[35] Weldesenbet H, Worku A, Shumbej TJBrn. Prevalence, infection intensity and associated factors of soil transmitted helminths among primary school children in Gurage zone, South Central Ethiopia: a cross-sectional study design. 2019;12(1):1–6. 10.1186/s13104-019-4254-8 PMC6469099 30992048

[36] Samuel F, Demsew A, Alem Y, Hailesilassie YJBPH. Soil transmitted Helminthiasis and associated risk factors among elementary school children in ambo town, western Ethiopia. 2017;17(1):1–7. 10.1186/s12889-017-4809-3 PMC5634961 29017470

[37] Yaro CA, Kogi E, Luka SA, Kabir JJDSAHMJ. School-based Cross-sectional Survey on Soil-transmitted Helminths in Rural Schools of Kogi East, Nigeria. 2020;2(1):10–9.

[38] Dejon-Agobé JC, Honkpehedji YJ, Zinsou JF, Edoa JR, Adégbitè BR, Mangaboula A, et al. Epidemiology of schistosomiasis and soil-transmitted helminth coinfections among schoolchildren living in Lambaréné, Gabon. 2020;103(1):325. 10.4269/ajtmh.19-0835 PMC7356410 32431272

[39] Nkouayep VR, Nejsum P, Cleopas DFD, Nadia NAC, Joël ATR, Mbida MJIJoTD, et al. Prevalence and Risk Factors of Infection with Soil Transmitted Helminths in Children from Bandjoun, the West Region of Cameroon. 2020:34–43. doi: 10.1155/2020/8832724 32963817 PMC7502121

[40] Dorkenoo MA, Agbeko F, Dokoto H, Plate D, Fiawoo M, Yakpa K, et al. Prevalence of Soil-Transmitted Helminths and Intestinal Protozoa among School Children in Lome, Togo. 2021;11(2):313–28.

[41] González Quiroz DJ, Agudelo Lopez SdP, Arango CM, Acosta JEO, Bello Parias LD, Alzate LU, et al. Prevalence of soil transmitted helminths in school-aged children, Colombia, 2012–2013. 2020;14(7):e0007613. 10.1371/journal.pntd.0007613 PMC7390406 32678821

[42] Rajan VXC, Sivamani M, Appalaraju BJTP. Prevalence and the factors influencing soil-transmitted helminths among school age children (5–14 years age) in a rural area of Coimbatore district. 2020;10(2):74. 10.4103/tp.TP_33_19 PMC7951075 33747872

[43] Kaliappan S, Ramanujam K, Manuel M, Farzana J, Janagaraj V, Laxmanan S, et al. Soil-transmitted helminth infections after mass drug administration for lymphatic filariasis in rural southern India. 2021. 10.1111/tmi.13697 34704320

[44] Laoraksawong P, Suntaraluk A, Kongnil W, Pongpanitanont P, Janwan PJIJoP. Prevalence of Soil–Transmitted Helminth Infections and Associated Risk Factors among Schoolchildren in Nakhon Si Thammarat, Thailand. 2020;15(3):440. 10.18502/ijpa.v15i3.4210 PMC7548470 33082810

[45] Crompton D, Savioli LJBotWHO. Intestinal parasitic infections and urbanization. 1993;71(1):1. PMC2393435 8440028

[46] Organization WH. Ending the neglect to attain the sustainable development goals: a road map for neglected tropical diseases 2021–2030: overview. 2020.

