# Supplementary material for: Prevalence, intensity of infection and associated risk factors of soil-transmitted helminth infections among school children at Tachgayint woreda, Northcentral Ethiopia
Source: PLoS One. 2022 Apr 8;17(4):e0266333. doi: 10.1371/journal.pone.0266333 (PMC8993015; doi:10.1371/journal.pone.0266333)
Supplement: S1 Table — (DOCX) [file pone.0266333.s001.docx]

**Prevalence of STH Species among school children of Tachgayint woreda, Northcentral Ethiopia.**

| **STH Infections** | **Frequency** | **Percentage** |
| --- | --- | --- |
| A. lumbricoides | 89 | 27.40 |
| Hookworm Species | 14 | 4.30 |
| T. trichiura | 10 | 3.10 |
| Mixed Infection | 4 | 1.20 |
| No Infection | 208 | 64.10 |
| Total | 325 | 100.0 |
